# Interlaboratory Development and Validation of a HRM Method Applied to the Detection of JAK2 Exon 12 Mutations in Polycythemia Vera Patients

**DOI:** 10.1371/journal.pone.0008893

**Published:** 2010-01-26

**Authors:** Valerie Ugo, Sylvie Tondeur, Marie-Laurence Menot, Nadine Bonnin, Gerald Le Gac, Carole Tonetti, Veronique Mansat-De Mas, Lydie Lecucq, Jean-Jacques Kiladjian, Christine Chomienne, Christine Dosquet, Nathalie Parquet, Luc Darnige, Marc Porneuf, Martine Escoffre-Barbe, Stephane Giraudier, Eric Delabesse, Bruno Cassinat, for the French Intergroup of Myeloproliferative disorders (FIM)

**Affiliations:** 1 CHU BREST, Laboratoire d'Hematologie, Brest, France; 2 CHU Montpellier, Laboratoire d'Hématologie, Hôpital Saint-Eloi, Montpellier, France; 3 AP-HP, Unite de Biologie Cellulaire, Hopital Saint-Louis, Paris, France; 4 INSERM U613, Brest, France; 5 AP-HP, Laboratoire d'Hematologie, Hopital Henri Mondor, Creteil, France; 6 CHU Toulouse, Laboratoire d'Hematologie, Hôpital Purpan, Toulouse, France; 7 AP-HP, Hematology Department, Hopital Avicenne, Bobigny, France; 8 AP-HP, Unite de Thérapie Cellulaire, Hopital Saint-Louis, Paris, France; 9 AP-HP, Laboratoire d'Hematologie, Hopital Europeen G. Pompidou, Paris, France; 10 CH Le Foll, Service d'Hématologie, Saint Brieuc, France; 11 CHU Rennes, Service d'Hematologie, Hopital Pontchaillou, Rennes, France; 12 PV-Nord Group, Hopital Saint-Louis, Paris, France; University of Barcelona, Spain

## Abstract

**Background:**

Myeloproliferative disorders are characterized by clonal expansion of normal mature blood cells. Acquired mutations giving rise to constitutive activation of the JAK2 tyrosine kinase has been shown to be present in the majority of patients. Since the demonstration that the V617F mutation in the exon 14 of the *JAK2* gene is present in about 90% of patients with Polycythemia Vera (PV), the detection of this mutation has become a key tool for the diagnosis of these patients. More recently, additional mutations in the exon 12 of the *JAK2* gene have been described in 5 to 10% of the patients with erythrocytosis. According to the updated WHO criteria the presence of these mutations should be looked for in PV patients with no JAK2 V617F mutation. Reliable and accurate methods dedicated to the detection of these highly variable mutations are therefore necessary.

**Methods/Findings:**

For these reasons we have defined the conditions of a High Resolution DNA Melting curve analysis (HRM) method able to detect JAK2 exon 12 mutations. After having validated that the method was able to detect mutated patients, we have verified that it gave reproducible results in repeated experiments, on DNA extracted from either total blood or purified granulocytes. This HRM assay was further validated using 8 samples bearing different mutant sequences in 4 different laboratories, on 3 different instruments.

**Conclusion:**

The assay we have developed is thus a valid method, adapted to routine detection of JAK2 exon 12 mutations with highly reproducible results.

## Introduction

Myeloproliferative disorders (MPDs) are hematological malignancies characterized by an accumulation of mature cells in the peripheral blood. Usually, Chronic Myeloid Leukemia (CML), a well characterized entity harbouring the recurrent t(9;22) translocation and the resulting *BCR-ABL1* fusion gene, is separated from the classical MPDs such as Polycythemia Vera (PV), Essential Thrombocytemia (ET) and Primary Myelofibrosis (PMF), in which a molecular abnormality has long time been ignored. In the latter group, the recurrent V617F mutation in the exon 14 of the *JAK2* gene has been identified in 2005 [1]–[4] and is currently a key marker for MPD diagnosis [5] as this mutation is present in 90%, 60% and 50% of PV, TE and PMF respectively [6]. In 2007, novel recurrent mutations clustered in a highly conserved region in exon 12 of the *JAK2* gene have been described in patients with PV or Idiopathic erythrocytosis [7]. Exon 14 and exon 12 mutations differ by 2 main characteristics: the V617F mutation is limited to only one base change (G1846T) found in all subtypes of MPDs [6] as well as in splanchnic vein thrombosis [8] and some myelodysplastic syndromes patients [9]. Exon 12 mutations on the other hands are extremely variable in sequence [10] and so far restricted to polycythaemia patients. In a recent study, using allele specific PCR, we reported the presence of JAK2 exon 12 mutations in 8 out of 24 PV patients negative for JAK2 V617F mutation, but failed to detect these mutations in patients with idiopathic erythrocytosis [11].

The detection of JAK2 exon 12 mutations is technically much more complicated than V617F mutation detection. Although some mutants are more frequent than others there has been an increasing number of different deletions, insertions or base changes described in the literature since the initial description [7]. Until recently, mutant detection had to be addressed either by direct sequencing (of low sensitivity) or allele specific PCR (of good sensitivity) but inadequate in a routine diagnosis setting with so many different mutations requiring multiple individual PCR reactions.

High Resolution DNA Melting curve analysis (HRM) is based on DNA melting in the presence of saturating DNA binding dyes. Sequence variants are inferred from changes in the melting transition of the PCR product as, depending on their GC content, length or sequence, different PCR products have different melting temperatures [12], whether mutated sequences are known or not. HRM methods have now been adapted to real-time PCR instruments and, compared to sequencing or AS-PCR, represent high throughput and time saving methods with the further advantage of reducing post-PCR handling of PCR products. HRM technology has been adapted to the identification of bacterial species [13] or subtypes [14], human SNP genotyping [15] or mutation detection [16], [17]. However, instruments vary widely in their ability to genotype variants by whole amplicon melting analysis [18], [19]. Similarly, several DNA binding dyes may be used with variable success [18], [19].

Because HRM technology could be a rapid and convenient tool for detecting the various JAK2 exon12 mutations, we decided to develop one such method with the prerequisite that it should be reliable enough to give similar results on 2 different instruments in 2 different sites. Then, the assay has been further validated on a cohort of 8 different mutants and 4 non mutated DNA in 2 additional centres, of whom one functioned in a blind manner.

## Materials and Methods

### Patients and Samples

In accordance with our recent results [11] and previous published studies [7] we tested patients presenting with erythrocytosis, low serum Epo levels and no V617F JAK2 mutation. The method was tested on samples from 39 patients from our previous study and 50 new patients addressed for diagnosis. DNA was extracted from total blood or granulocytes. The local institutional ethics committee “Comite Consultatif de Protection des Personnes dans la Recherche Biomedicale” approved the study, and all patients gave written informed consent.

### HRM Method

Three instruments were used in 4 different French centres: a LighCycler 480 (Roche Applied Sciences) in Toulouse and Brest, an ABI 7500 fast (AppliedBiosystems) in Paris and an ABI 7900 (AppliedBiosystems) in Creteil. PCR reactions were performed in a 12 µl final volume containing 20 ng of genomic DNA and 0.2 µM of forward (5′-ACCAACCTCACCAACATTACAGAG-3′) and reverse (5′-AAAAGGACAAAAAAGACAGTAATGAGTATC-3′) primers defining a 184 bp amplicon. When LightCycler apparatus was used, the LC480 HRM master mix (Roche), containing Resolight© as DNA binding dye, was used and 3 mM MgCl2 was added, whereas the AmpliTaq Gold PCR Master Mix (AppliedBiosystems) with 1.5 µM of Syto-9 (Invitrogen) were used with the ABI 7500 fast or ABI 7900 instruments. Amplification was performed by 50 cycles of 95°C for 15 secs, 63°C for 15 secs and 72°C for 25 secs followed by a melt according to each manufacturer instructions.

### Sequencing Analysis

PCR reactions were performed in a 20 µL reaction volume containing the following: 10 pmol of each primer forward primer was 5′-CTCCTCTTTGGAGCAATTCA-3′ and reverse primer was 5′-GAGAACTTGGGAGTTGCGATA-3′, 1x PCR buffer (Qiagen), 200 µM each dNTPS (Invitrogen), 1U of HotStarTaq DNA polymerase (Qiagen) and 20 ng of DNA. Cycling conditions were as follows: 95°C for 15 min, 35 cycles of 94°C for 20 s, 59°C for 20 sec, 72°C for 45 s followed by a final elongation step at 72°C for 10 min. PCR Amplified fragment was 495 bp in length. Sequencing analyses were performed using a fluorescent-tagged dideoxy chain termination method with a 3130XL-DNA sequencing system (Applied Biosystem).

## Results

### HRM Analysis Is a Suitable Method for JAK2 Exon 12 Mutations Detection

**Figure 1:**
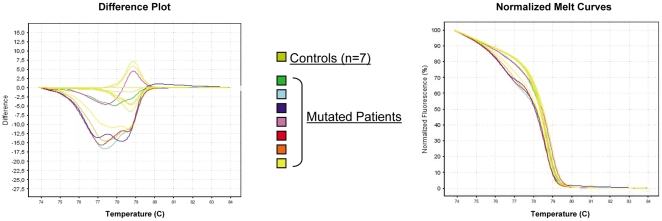
HRM analysis. HRM profiles of 7 patients known to harbor JAK2 exon 12 mutations compared to 7 controls. Analysis of total blood DNA on the ABI7500 fast instrument.

We previously reported several JAK2 V617F negative polycythemia patients with JAK2 exon 12 mutations detected by allele specific Polymerase Chain Reaction (AS-PCR) [11]. In the present study we first used total blood DNA from these positive patients in order to set up a new assay using HRM technology in 2 centres. The assay was first developed on a Lightcycler 480 (Roche) in Toulouse, and then tested on an ABI 7500 fast (AppliedBiosystems) instrument in Paris. Using HRM method allowed to identify every patient known to be mutated, whereas all control patients showed a wild type profile (Figure 1). These results confirmed that High Resolution DNA Melting curve analysis is a suitable method for the detection of JAK2 exon 12 abnormalities.

In order to test for the reproducibility of the method we have analysed 2 positive samples characterized by different mutant sequences in 3 independent experiments, each performed at 1 week interval. Results were highly similar in all three experiments (not shown), proving that the HRM method tested herein could have sufficient robustness for a diagnostic purpose.

**Figure 2:**
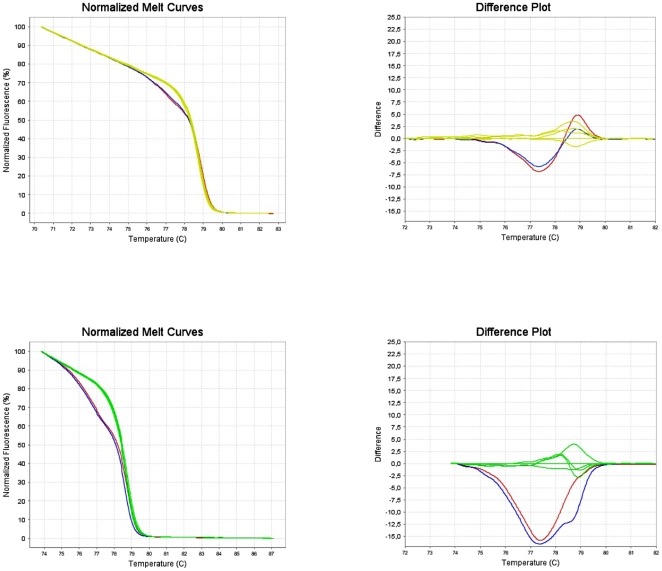
Comparison between total blood and granulocyte DNA. HRM analysis performed in 2 patients on DNA extracted from total blood or purified granulocytes from the same sample. Granulocytes in red, Blood in blue, normal controls in green. Patient 1 in upper panel; Patient 2 in lower panel.

Though it is now widely admitted that JAK2 V617F mutations can be detected with similar efficacy in DNA from peripheral blood or purified granulocytes, this has not been extensively studied for the JAK2 exon 12 mutations. We compared in 2 patients the results obtained with DNA extracted from total blood or from purified granulocytes. Similar results were observed whatever the source of DNA (Figure 2).

### Applications of the HRM Analysis

Samples (n = 39) of patients with either idiopathic erythrocytosis or PV from our previous report [11] which were negative by AS-PCR for exon 12 mutations were analysed by the HRM method and were again found negative. In the analysis of new patients addressed to our laboratories for MPD diagnosis, 9 out of 35 patients presenting with an increased hematocrit, low Epo levels and absence of the JAK2 V617F mutation, harboured a mutation confirmed by sequencing analysis.

### Validation of the Method in 2 Additional Centres

It has been reported that important discrepancies could be observed when one HRM method was used on different instruments [18], [19]. In a preliminary approach, we thus compared the diagnostic accuracy of our method by analysing the 9 positive samples on 2 instruments, in 2 separate laboratories in Toulouse and Paris. Each laboratory used different DNA binding dyes (Resolight© on the LC480 instrument and Syto-9 on the ABI 7500 instrument) also reported to be source for discrepancies [18], [19]. Although shifting temperatures and curves shapes were somewhat different between the two instruments, results were undoubtedly similar in their interpretation.

**Figure 3:**
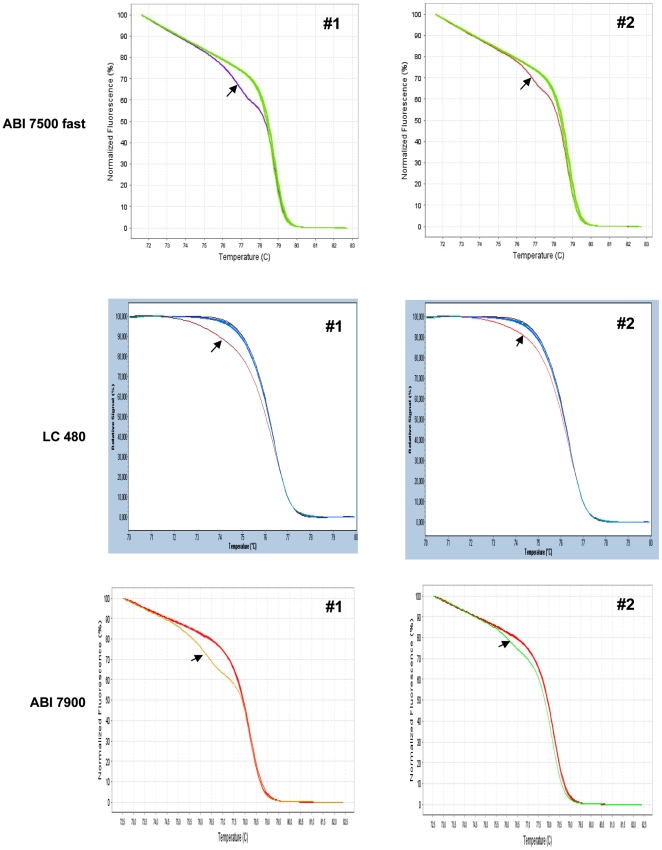
HRM profiles on 3 different instruments for 2 patients. Total blood DNA from 2 patients was analysed on 3 different instruments together with non mutated DNA. Arrows show mutant DNA.

The validation of the method was completed by analysing 8 samples bearing different mutated sequences (clinical data and mutant subtypes are given in Table 1) in 2 additional laboratories, Brest and Creteil, which had not participated in the set up of the HRM assay. In one of the two labs (Creteil), the analysis was performed in a completely blind manner, on12 anonymous DNA samples (8 mutated and 4 wild type) on an ABI 7900 instrument (AppliedBiosystems) wheras a LC480 instrument (Roche) was used in Brest. The HRM method was equally able to discriminate mutant from wild type samples on the 3 different instruments tested (Figure 3 represents an example of the comparative results obtained on different instruments). Both laboratories perfectly identified every mutated sample, without any change in the experimental protocol confirming this HRM method as a robust and efficient method even in an inter-laboratory fashion.

**Table 1:** Clinical and biological data of 8 patients with exon 12 mutations tested in 4 laboratories (RCM: Red cell mass. ND: Not done).

N°	Age (Y)	Sex	RCM (% excess)	Hematocrit (%)	WBC (x10^9^/L)	Platelets (x10^9^/L)	Epo	EECs	Spleen	Mutation
1	61	F	137	63	17	162	low	ND	N	N542-E543del
2	66	F	68	62	5,8	274	low	Yes	N	H538Q-K539L
3	52	M	ND	67	12,5	260	low	Yes	N	H538-K539delinsL
4	38	M	ND	64	11,6	354	low	ND	N	K539L
5	71	F	68	72	7,9	151	low	ND	Y	I546_F547ins11
6	57	M	141	62	12,8	259	low	ND	N	R541_E543delinsK
7	71	M	113	53	16,3	1425	ND	ND	N	E543_D544del
8	80	F	57	57	5,6	378	low	Yes	N	I540_N542delinsK

## Discussion

As WHO criteria for Polycythaemia Vera diagnosis include the “presence of JAK2 V617F or similar mutation„ [5], detection of JAK2 exon 12 mutations is of importance for the 5 to 10% of patients presenting with a suspicion of PV but with no JAK2 V617F mutation. Furthermore, pharmacological JAK2 inhibitors are, at least for some of them, equally active on V617F and JAK2 exon 12 mutations [20], reinforcing the need for a convenient tool to detect those mutations.

Several different mutations are now described [10] in the JAK2 exon 12 clustered in a short region of about 35 bases [7], easily studied in only one round of PCR. HRM analysis, based on the detection of a difference in fusion temperature or a difference in the shape of the fusion curve, is able to detect variations in the GC content, the sequence or the length of PCR products. It has recently been successfully adapted to detect JAK2 exon 12 mutations [21], [22]. However, one difficulty in the field of HRM is the possibility to transfer one method from one instrument to another, because variations have been reported in comparative studies [18], [19]. For these reasons, once having defined an HRM method which may be able to detect patients known to present JAK2 exon 12 mutations, we validated the method as reproducible and transferable on three different instruments using different DNA binding dyes. The method allowed to identify mutant patients which, as previously reported for exon 12 mutated patients, presented with erythrocytosis and a low circulating erythropoietin level, high WBC in the majority of them but normal platelet counts. Similarly to the JAK2 V617F mutation [23], exon 12 mutations can be found in all lineages, although T lymphocytes are frequently found negatives [24]. It is thus not surprising that similar results can be obtained from DNA extracted from total blood or purified granulocytes. These results suggest that, at least using HRM methods, the detection of JAK2 exon 12 mutations for MPD diagnosis may be performed with similar efficiency on DNA from total blood or purified granulocytes. Notably, we have reanalysed several DNA samples from patients previously found not mutated using the AS-PCR approach, but failed to find any additional mutated patient. Nevertheless, the HRM method presented in this study is able to detect more mutations than AS-PCR, is a high throughput method and much more suitable for routine laboratory diagnosis, and is inter-laboratory and inter-instrument reproducible.

## References

[1] James C, Ugo V, Le Couédic JP, Staerk J, Delhommeau F (2005). A unique clonal JAK2 mutation leading to constitutive signalling causes polycythaemia vera.. Nature.

[2] Baxter EJ, Scott LM, Campbell PJ, East C, Fourouclas N (2005). Acquired mutation of the tyrosine kinase JAK2 in human myeloproliferative disorders.. Lancet.

[3] Kralovics R, Passamonti F, Buser AS, Teo SS, Tiedt R (2005). A gain-of-function mutation of JAK2 in myeloproliferative disorders.. New Engl J Med.

[4] Levine RL, Wadleigh M, Cools J, Ebert BL, Wernig G (2005). Activating mutation in the tyrosine kinase JAK2 in polycythemia vera, essential thrombocythemia, and myeloid metaplasia with myelofibrosis.. Cancer Cell.

[5] Tefferi A, Vardiman JW (2008). Classification and diagnosis of myeloproliferative neoplasms: the 2008 World Health Organization criteria and point-of-care diagnostic algorithms.. Leukemia.

[6] Campbell PJ, Green AR (2006). The myeloproliferative disorders.. New Engl J Med.

[7] Scott LM, Tong W, Levine RL, Scott MA, Beer PA (2007). JAK2 exon 12 mutations in polycythemia vera and idiopathic erythrocytosis.. New Engl J Med.

[8] Kiladjian JJ, Cervantes F, Leebeek FW, Marzac C, Cassinat B (2008). The impact of JAK2 and MPL mutations on diagnosis and prognosis of splanchnic vein thrombosis: a report on 241 cases.. Blood.

[9] Steensma DP, Dewald GW, Lasho TL, Powell HL, McClure RF (2005). The JAK2 V617F activating tyrosine kinase mutation is an infrequent event in both “atypical” myeloproliferative disorders and myelodysplastic syndromes.. Blood.

[10] Pietra D, Li S, Brisci A, Passamonti F, Rumi E (2008). Somatic mutations of JAK2 exon 12 in patients with JAK2 (V617F)-negative myeloproliferative disorders.. Blood.

[11] Kouroupi E, Zoi K, Parquet N, Zoi C, Kiladjian JJ (2008). Mutations in exon 12 of JAK2 are mainly found in JAK2 V617F-negative polycythaemia vera patients.. Brit J Haematol.

[12] Ririe KM, Rasmussen RP, Wittwer CT (1997). Product differentiation by analysis of DNA melting curves during the polymerase chain reaction.. Anal Biochem.

[13] Cheng JC, Huang CL, Lin CC, Chen CC, Chang YC (2006). Rapid detection and identification of clinically important bacteria by high-resolution melting analysis after broad-range ribosomal RNA real-time PCR.. Clin Chem.

[14] Lin JH, Tseng CP, Chen YJ, Lin CY, Chang SS (2008). Rapid differentiation of influenza A virus subtypes and genetic screening for virus variants by high-resolution melting analysis.. J Clin Microbiol.

[15] Seipp MT, Pattison D, Durtschi JD, Jama M, Voelkerding KV (2008). Quadruplex genotyping of F5, F2, and MTHFR variants in a single closed tube by high-resolution amplicon melting.. Clin Chem.

[16] Pichler M, Balic M, Stadelmeyer E, Ausch C, Wild M (2009). Evaluation of high-resolution melting analysis as a diagnostic tool to detect the BRAF V600E mutation in colorectal tumors.. J Mol Diag.

[17] Holden JA, Willmore-Payne C, Coppola D, Garrett CR, Layfield LJ (2007). High-resolution melting amplicon analysis as a method to detect c-kit and platelet-derived growth factor receptor alpha activating mutations in gastrointestinal stromal tumors.. Am J Clin Pathol.

[18] Herrmann MG, Durtschi JD, Bromley LK, Wittwer CT, Voelkerding KV (2006). Amplicon DNA melting analysis for mutation scanning and genotyping: cross-platform comparison of instruments and dyes.. Clin Chem.

[19] Herrmann MG, Durtschi JD, Wittwer CT, Voelkerding KV (2007). Expanded instrument comparison of amplicon DNA melting analysis for mutation scanning and genotyping.. Clin Chem.

[20] Lasho TL, Tefferi A, Hood JD, Verstovsek S, Gilliland DG (2008). TG101348, a JAK2-selective antagonist, inhibits primary hematopoietic cells derived from myeloproliferative disorder patients with JAK2V617F, MPLW515K or JAK2 exon 12 mutations as well as mutation negative patients.. Leukemia.

[21] Jones AV, Cross NC, White HE, Green AR, Scott LM (2008). Rapid identification of JAK2 exon 12 mutations using high resolution melting analysis.. Haematologica.

[22] Rapado I, Grande S, Albizua E, Ayala R, Hernández JA (2009). High resolution melting analysis for JAK2 Exon 14 and Exon 12 mutations: a diagnostic tool for myeloproliferative neoplasms.. J Mol Diag.

[23] Delhommeau F, Dupont S, Tonetti C, Massé A, Godin I (2007). Evidence that the JAK2 G1849T (V617F) mutation occurs in a lymphomyeloid progenitor in polycythemia vera and idiopathic myelofibrosis.. Blood.

[24] Li S, Kralovics R, De Libero G, Theocharides A, Gisslinger H (2008). Clonal heterogeneity in polycythemia vera patients with JAK2 exon12 and JAK2-V617F mutations.. Blood.

